# Functional Connectivity of Language Regions of Stroke Patients with Expressive Aphasia During Real-Time Functional Magnetic Resonance Imaging Based Neurofeedback

**DOI:** 10.1089/brain.2019.0674

**Published:** 2019-10-17

**Authors:** Sujesh Sreedharan, KM Arun, PN Sylaja, Chandrasekharan Kesavadas, Ranganatha Sitaram

**Affiliations:** ^1^ Division of Artificial Internal Organs, Department of Medical Devices Engineering, Biomedical Technology Wing, Sree Chitra Tirunal Institute for Medical Sciences and Technology (SCTIMST), Trivandrum, India.; ^2^ Department of Imaging Sciences and Intervention Radiology, Sree Chitra Tirunal Institute for Medical Sciences and Technology (SCTIMST), Trivandrum, India.; ^3^ Department of Neurology, Sree Chitra Tirunal Institute for Medical Sciences and Technology (SCTIMST), Trivandrum, India.; ^4^ Institute for Biological and Medical Engineering, Center for Brain-Machine Interfaces and Neuromodulation, and Department of Psychiatry and Division of Neuroscience, Faculties of Engineering, Biology and Medicine, Pontificia Universidad Católica de Chile, Santiago, Chile.

**Keywords:** aphasia, functional connectivity, neurofeedback, real-time fMRI, self-regulation, stroke

## Abstract

Stroke lesions in the language centers of the brain impair the language areas and their connectivity. This article describes the dynamics of functional connectivity (FC) of language areas (FCL) during real-time functional magnetic resonance imaging (RT-fMRI)-based neurofeedback training for poststroke patients with expressive aphasia. The hypothesis is that FCL increases during the upregulation of language areas during neurofeedback training and that the training improves FCL with an increasing number of sessions and restores it toward normalcy. Four test and four control patients with expressive aphasia were recruited for the study along with four healthy volunteers termed as the normal group. The test and normal groups were administered four neurofeedback training sessions in between two test sessions, whereas the control group underwent only the two test sessions. The training session requires the subject to exercise language activity covertly so that it upregulates the feedback signal obtained from the Broca's area (in left inferior frontal gyrus) and amplifies the feedback when it is correlated with the Wernicke's area (in left superior temporal gyrus) using RT-fMRI. FC was measured by Pearson's correlation coefficient. The results indicate that the FC of the test group was weaker in the left hemisphere than that of the normal group, and post-training the connections have strengthened (correlation coefficient increases) in the left hemisphere when compared with the control group. The connections of language areas strengthened in both hemispheres during neurofeedback-based upregulation, and multiple training sessions strengthened new pathways and restored left hemispheric connections toward normalcy.

## Introduction

Aphasia or loss of speech is the most prevalent disability in stroke survivors. Stroke lesions affecting the Broca's area (inferior frontal gyrus or IFG), Wernicke's area (superior temporal gyrus or STG) and connecting white matter tracts, can lead to aphasia. Accordingly, aphasia can be broadly classified as Broca's aphasia (failure to express language), Wernicke's aphasia (failure to comprehend language), or conduction aphasia (Dronkers and Baldo, 2010). In Broca's aphasia or expressive aphasia, the expression of speech is reduced and is limited to short sentences of very few words and is also referred to as telegraphic speech. The linking of words to form sentences is severely affected and agrammatical. Vocabulary access is limited, and speech generation is laborious and nonfluent. The person may comprehend speech relatively well and also be able to read well; however, the ability to write is limited. Lesions in the Broca's area in the IFG, the lower part of the precentral gyrus, and the opercular and insular regions are associated with naming difficulties and overall expressive language deficits in individuals with Broca's aphasia (Hojo et al., 1985; Plowman et al., 2012).

Subacute and chronic recovery is thought to be caused by brain plasticity, wherein either the ipsilesional areas or the contralateral homotopic regions take over the function from the damaged core (Crosson et al., 2007). This neuroplasticity may also accompany a change in the functional connectivity (FC), recruiting a different set of brain regions when compared with the original FC network. FC is defined as a statistical dependence between the neural signals in two brain regions (Friston, 1994; Rubinov and Sporns, 2010). Stroke lesions could also damage the neural connection between brain regions and may result in FC changes among these areas (Grefkes and Fink, 2014).

Self-regulation of activity in specific areas of the brain is a promising tool for neurorehabilitation (Sitaram et al., 2016; Watanabe et al., 2017). Patients with compromised brain function, such as due to stroke, have been trained on real-time functional magnetic resonance imaging (RT-fMRI) for the regulation of activity in affected areas or surrounding areas to study the improvement of brain function (Cohen Kadosh et al., 2016; Emmert et al., 2017; Sorger et al., 2016; Young et al., 2017). “Operant conditioning” typically involves the presentation of rewards (or punishments) contingent upon a specific behavior of the organism. Conditioning takes place when the probability of an organism making a response has been modified by the contingency. RT-fMRI generates feedback of blood-oxygen-level-dependent (BOLD) activity from specific regions of the brain (Weiskopf et al., 2007).

The language function is carried out by the temporofrontal network in the brain with lateralization majorly to the left hemisphere, and either bilateral or right hemispheric involvement to a lesser extent (Pujol et al., 1999; Smitha et al., 2017). The arcuate fasciculus structurally connects Broca's and Wernicke's areas and is an important language pathway (Breier et al., 2008; Catani et al., 2005). An indirect pathway between the Wernicke's and Broca's areas through the inferior parietal cortex has been reported (Pujol et al., 1999). Ventral pathways connecting the anterior portion of the IFG and the temporal cortex through the extreme fiber capsule system, and the frontal operculum to the anterior temporal cortex through the uncinate fasciculus have been found (Friederici and Gierhan, 2013; Kümmerer et al., 2013). Stroke lesions not only affect the functioning of the lesioned brain region but also suppress activity in the interconnected regions that depend on excitatory inputs from the lesioned region (Grefkes and Fink, 2014). Based on the mentioned findings, the language network has been defined to include regions of the supraparietal (SP) cortex, the central opercular (CO) cortex, temporal pole, and frontal pole in addition to the Broca's area and the Wernicke's area.

One mode of recovery has been hypothesized as due to reactivation of deafferented brain regions through alternative pathways. Another hypothesis is that intact regions take over function from the infarcted regions. During animal studies, the formation of new synapses and axonal sprouting has been observed in the peri-infarcted cortex immediately after stroke and hypothesized as a spontaneous recovery mechanism (Grefkes and Fink, 2014). The mentioned recovery modes could result in new or stronger pathways (connections) in the FC network. Several studies have shown that left hemispheric perilesional involvement promotes better recovery, and right hemisphere involvement leads to poor recovery of language function. It has been hypothesized before that the right hemispheric recruitment is due to loss of inhibition by the left hemisphere rather than due to language recovery. As contrasting evidence, right hemispheric involvement has been shown to be important for language activation of healthy volunteers. This gives plausibility to the hypothesis that in the event of left hemispheric stroke, these right brain regions may take over the language function and promote recovery. Several studies have reported such right brain activation or bilateral activation during language tasks after stroke (Crosson et al., 2007; Thompson and den Ouden, 2008).

In a study by Warren et al. (2009), aphasic patients demonstrated a selective disruption of the normal FC between left and right anterolateral superior temporal cortices. In another study of FC changes in the left frontoparietal network (LFPN) of aphasic patients, reduced FC between the LFPN and the right middle frontal cortex, medial frontal cortex, and right inferior frontal cortex was found in aphasic patients as compared with that of controls (Zhu et al., 2014). In a study by Sandberg et al. (2015), direct training effects coincided with increased FC for regions involved in abstract word processing and generalization effects coincided with increased FC for regions involved in concrete word processing. Another study in early stroke patients without clinically documented language deficits showed decreased resting state FC in the language network and verbal fluency deficits (Nair et al., 2015). In a study of individual anatomical whole-brain connectomes from 90 left hemisphere stroke survivors using diffusion magnetic resonance (MR) images, the modularity of the residual white matter network organization, the probability of brain regions clustering together, and the degree of fragmentation of left hemisphere networks were studied. Greater poststroke left hemisphere network fragmentation and higher modularity index were associated with more severe chronic aphasia, controlling for the size of the stroke lesion. Even when the left hemisphere was relatively spared, subjects with disorganized community structure had significantly worse aphasia, particularly when key temporal lobe regions were isolated into segregated modules. These results suggest that white matter integrity and disorganization of neuronal networks could be important determinants of chronic aphasia severity (Marebwa et al., 2017).

In this study, we have used RT-fMRI as a neurofeedback training strategy to improve neural activation in the language areas as well as their FC in poststroke patients with expressive aphasia. The changes in the FC are assessed by partitioning the language network into six modules in each hemisphere and assessing the intermodular connectivity changes. The six modules correspond to the (1) Broca's area and adjoining frontal regions, (2) the Wernicke's area and adjoining temporal regions, and parts of the (3) SP cortex, (4) the CO cortex, (5) the frontal pole, and (6) the temporal pole.

### Objectives of the study

The objectives of this article were to study (1) whether RT-fMRI-based neurofeedback training improves the FC of language areas (FCL) in the brain, (2) the effect of upregulation during the neurofeedback training on the FCL, and (3) how the FCL differs between the test and normal groups, and whether the training reduces this difference with sessions.

The hypothesis is that FCL increases during the upregulation of language areas during the neurofeedback training and that the training improves FCL between frontal and temporal regions in the left perisylvian cortex with an increasing number of sessions and restores it toward normalcy.

## Methodology

In this study, we recruited four test patients and four control patients, during a period between 6 weeks and 6 months poststroke. The patients were diagnosed with expressive aphasia (Broca's) only, and their language comprehension was relatively preserved. In addition, a group of four healthy volunteers (normal group) participated in the study. The study protocol was approved by the ethics committee of Sree Chitra Tirunal Institute for Medical Sciences and Technology (SCTIMST) and written informed consent was obtained from each patient before the study. The detailed description of the methodology including patient selection, administration of neurofeedback training and behavioral tests, BOLD activation in language areas of the brain, and the language performance has been reported in an earlier publication (Sreedharan et al., 2019).

Patients with age >18 years, diagnosed with expressive aphasia, and within an interval of 6 weeks to 6 months poststroke were recruited for the study. Once the patients were recruited, six RT-fMRI sessions were planned for test patients and two for the control patients. The patients were given instructions for differentiating the rest block and the upregulation block, as well as to respond to a picture-naming task with an appropriate button press. They were also advised to use a suitable strategy for upregulation of language to raise the activation levels displayed on-screen during scanning, such as making a speech, having a conversation, reciting a poem, or any other form of language activity. These tasks were instructed to be performed covertly without any head motion. The stroke lesions were majorly affecting the patients in the left hemisphere and resulting in motor deficits of right upper limb as well as right lower limb weakness, along with language deficit of either slurring of speech or inability to speak. RT-fMRI sessions were conducted on 6 days in the test and normal groups, with a gap of roughly 1 week in between consecutive sessions. The control group was not provided noncontingent feedback or sham feedback for ethical reasons. The effectiveness of RT-fMRI neurofeedback to achieve self-regulation with the use of controls who were given sham feedback has already been reported (Caria et al., 2011; deCharms et al., 2004).

In the first and last sessions, picture-naming tasks were administered after each baseline block and upregulation block. Preprocessing of data was done using Statistical Parametric Mapping (SPM; www.fil.ion.ucl.ac.uk/spm) toolbox. Preprocessing involved realignment, coregistration of anatomical and functional scans, and normalization and smoothing using standard procedures in SPM (Friston et al., 1994). The normalized functional scans had an isometric voxel size of 3 mm, whereas the normalized anatomical scan had a voxel size of [1.0, 1.0, 1.1] mm. The smoothing was performed by convolving with a Gaussian kernel of 6 mm full width at half of maximum. The preprocessed data were then analyzed for FC using the CONN17 toolbox (Whitfield-Gabrieli and Nieto-Castanon, 2012).

### Real-time fMRI and neurofeedback

An MR scanner (Siemens Avanto) of 1.5T field strength was used to acquire fMRI signals using echo planar imaging (EPI) sequences. The EPI was acquired with 16 slices of 64 × 64 pixels in a single repetition time of 1.5 s and echo time of 45 ms. A high-resolution structural image was acquired before the fMRI sessions to overlay the functional maps on the brain structure. Each scan was exported from the MR workstation after reconstruction to the Turbo Brain Voyager (TBV) computer. To enable this in the Siemens MR system, the configuration was set by a user interface called the *Ideacmdtool*. Feedback was visually presented in the form of a thermometer during the upregulation block. During the baseline block, feedback was not provided, and the thermometer was displayed at a constant level of 10 blue colored bars. An increase in the feedback was shown as red bars in the thermometer, and for any reduction below baseline, the blue levels were proportionately removed.

TBV functioned as the core of the neurofeedback loop. The MR images acquired were corrected for head motion artifacts. In addition, spatial smoothing was performed to reduce the effect of noise. Neurofeedback was provided from two clusters of activation or regions of interest (ROIs), in and around Broca's area (ROI_1_) and Wernicke's area (ROI_2_) identified with the help of a functional localizer task. The localizer had a sequence of five blocks of word generation tasks interspersed with rest blocks of equal duration, each task consisting of a letter from the Malayalam alphabet presented visually. The functional localizer was processed by the TBV in real time, and the significantly activated clusters were generated immediately after the localizer run. The details of the ROIs selected are given as Supplementary Data.

### Feedback computation

To compute the feedback value (BF), our custom-built Matlab script used the mean of the latest three BOLD activity levels from the first ROI, and latest 10 values from the time series of the two ROIs to compute the correlation coefficient (as a measure of FC) after the nth volume based on the equation:

\begin{align*}
BF \left( n \right) = mean \left( {RO{I_1}} \right) { \rm{*}} \left[ {1 + corr \left( {RO{I_1} , \;RO{I_2}} \right) } \right] \tag{1}
\end{align*}

The BOLD feedback was presented only during the upregulation blocks and sequences ROI_1_ and ROI_2_ were corrected for the mean baseline activation from the previous baseline block. The correlation coefficient term is a measure of the FC among Broca's area and Wernicke's area and thereby serves to amplify the feedback value if a positive correlation is present and *vice versa*.

### Functional connectivity

The FC of the brain was analyzed using the CONN toolbox (www.nitrc.org/projects/conn). The subjects were grouped into the normal group, test group, and control group by specifying subject covariates. Each normal and test subject had 24 fMRI runs (6 sessions with 4 runs per session) and each control subject had 8 runs (2 sessions with 4 runs per session). The experimental conditions for within-subject effects were sessions S1 to S6: spanning the four runs of an RT-fMRI session, entire series (ES): spanning all the runs of all sessions, and baseline (BL), upregulation (UR), postbaseline test, and postupregulation test conditions as was defined in the RT-fMRI protocol. The entire series that incorporates all the scans of each session for a single subject was termed the ES condition. The temporal confounding factors were derived from all the modeled conditions as well as motion regressors obtained during the realignment step during preprocessing. The CompCor algorithm was used to regress out the effects of confounding factors, which consisted of the principal components of the gray matter, white matter, and cerebrospinal fluid regions of the subject's brain, modeled conditions and their time derivatives, and motion regressors (Whitfield-Gabrieli and Nieto-Castanon, 2012). The residual BOLD time series is used further for estimating the connectivity.

The ROIs chosen for the study included Broca's and Wernicke's areas and their right homologs, and 11 neighboring ROIs, 6 near the Broca's area and 5 near the Wernicke's area (restricted to each patient's active region during a *t*-statistic test of *p* < 0.01 for upregulation task in SPM). In addition, 50 ROIs from the automated anatomical landmarks (AALs) space (Tzourio-Mazoyer et al., 2002) known to be involved in language processing were also selected. These ROIs were grouped into modules as described in Tables 1 and 2.

**Table 1.** Regions of Interest Used for Connectivity Analysis Grouped into Modules: Left Hemisphere

*Left hemisphere*		
FL	Frontal language	Broca_L^*^, IFG triangularis, IFG operculum, frontal Mid_L^*^, SFG, frontal operculum (FO), MFG. [FL-1 to FL-7]
TL	Temporal language	pMTG, Wernicke_L^*^, angular gyrus (AG), toMTG, pSTG, TemporalMid_L^*^, pSMG, angular_L^*^. [TL-1 to TL-8]
CO	Central opercular	Insula_L^*^, central operculum (CO), insula, parietal operculum (PO), planum polare (PP), Heschl's gyrus (HG), planum temporale (PT), Heschl_L^*^, Rolandic Oper_L^*^ [CO-1 to CO-9]
FP	Frontal polar	Frontal pole (FP), frontal InfOrb_L^*^, frontal MidOrb_L^*^, frontal orbitalis (FO) [FP-1 to FP-4]
TP	Temporal polar	aSTG, temporal pole (TP), aMTG [TP-1 to TP-3]
SP	Supraparietal	SPL, postcentral gyrus (PostCG), precentral_L^*^, precentral gyrus (PreCG), supramarginal_L^*^, aSMG, postcentral_L^*^ [SP-1 to SP-7]

^*^ Patient-specific ROIs.

aXXX, anterior part of XXX; IFG, inferior frontal gyrus; MFG, middle frontal gyrus; MTG, middle temporal gyrus; pXXX, posterior part of XXX; SFG, superior frontal gyrus; SMG, supramarginal gyrus; SPL, supraparietal lobule; STG, superior temporal gyrus.

**Table 2.** Regions of Interest Used for Connectivity Analysis Grouped into Modules: Right Hemisphere

*Right hemisphere*		
rFL	Right frontal language	IFGoper.r, Broca_R^*^, SFG.r, FO.r, MFG.r, IFGtri.r [rFL-1 to rFL-6]
rTL	Right temporal language	pSTG.r, toMTG.r, Wernicke_R^*^, pSMG.r, pMTG.r, AG.r [rTL-1 to rTL-6]
rCO	Right central opercular	Insula_R, HG.r, PP.r, PT.r, PO.r, CO.r [rCO-1 to rCO-6]
rFP	Right frontal polar	Right frontal pole (FP.r), right frontal orbitalis (FO.r) [rFP-1 to rFP-2]
rTP	Right temporal polar	aSTG.r, right temporal pole, aMTG.r [rTP-1 to rTP-3]
rSP	Right supraparietal	PostCG.r, aSMG.r, PreCG.r, SPL.r [rSP-1 to rSP-4]

^*^ Patient-specific ROIs.

The first level of connectivity analysis was performed with all the mentioned 65 ROIs. This computes the FC between each pair of ROIs during each of the conditions specified for each of the subjects. The default setting of weighting the ROI time series with the hemodynamic response function-convolved blocks of each condition was used for computing the ROI-to-ROI connectivity during each of the conditions. The bandpass filter setting was in the frequency range from 0.008 to 0.09 Hz. In the second level analysis, several analysis of variances were performed in CONN with between-subject group contrasts and between condition contrasts of the computed ROI-to-ROI connections. The measure used for FC was the bivariate correlation, which is also known as the Pearson's correlation coefficient as shown in Equation 2 for two ROIs' time series *x* and *y*. This correlation coefficient was Fisher transformed to improve the normality assumptions of the data for further second-level generalized linear model analysis (Whitfield-Gabrieli and Nieto-Castanon, 2012).

\begin{align*}
corr \left( {x , y} \right) = {x^t}y / \left\{ { \parallel x \parallel . \parallel y \parallel } \right\} . \tag{2}
\end{align*}

### Modularity of the connectivity matrix

**FIG. 1.**
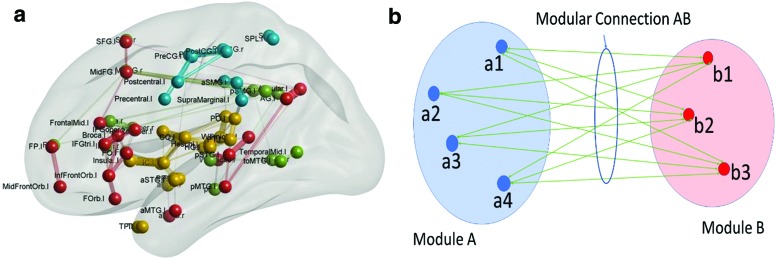
**(a)** Modular subnetworks for normal group. **(b)** FC between modules. Modular subnetworks for normal group and modular connections. Modular connection AB is found by summing the connection strengths between each ROI in module A to each ROI in module B, that is, CONN **(A, B)** = sum(CONN**(a_i_, b_j_)**) over all *i* and *j*. aXXX, anterior part of XXX; FC, functional connectivity; IFG, inferior frontal gyrus; MFG, middle frontal gyrus; MTG, middle temporal gyrus; pXXX, posterior part of XXX; ROIs, regions of interest; SFG, superior frontal gyrus; SMG, supramarginal gyrus; SPL, supraparietal lobule; STG, superior temporal gyrus. Color images are available online.

The connectivity matrix obtained for the normal group was first analyzed for modularity and split into modules in the left hemisphere as shown in Figure 1. Modularity is a statistic that quantifies the degree to which a network may be subdivided into nonoverlapping groups of nodes. Modules are densely connected groups of nodes in the FC network having only sparse interconnections between the modules (Newman, 2006). Both the number of modules and their extent are found by data-driven algorithms (Newman, 2006). In this study, modularity was analyzed using the Louvain algorithm (Blondel et al., 2008) implemented in the Brain Connectivity Toolbox (Rubinov and Sporns, 2010). Since this is a data-driven approach, varying partitions were obtained during each run of the algorithm on the normal data set. A partitioning was chosen that appeared more frequently during the partitioning runs, which divides the 65 ROIs into 4 modules; the partitions can fairly be considered as the frontotemporal, central opercular, posterior temporal, and parietal cortices.

**FIG. 2.**
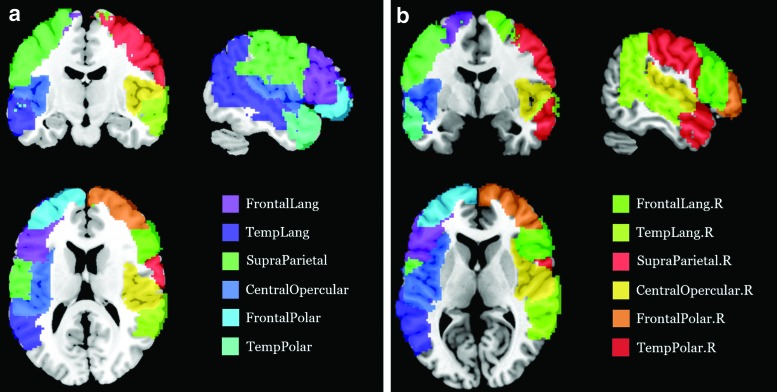
**(a)** Left hemisphere **(b)** right hemisphere. Modular subnetworks of the language areas for the left and right hemispheres of the brain. The colored regions consist of several ROIs from the automated anatomical landmarks space. Color images are available online.

The first module, extending mainly in the frontotemporal language network, was split based on anatomical location into three modules, which are termed the frontal polar (FP), frontal language (FL), and the temporal language (TL) modules. The second module in the CO region was split into the CO module and temporal polar (TP) module. One ROI (aMTG falling in the first module) was regrouped into the TP module based on the anatomical location rather than the modular structure. The third module was grouped with the TL module due to its location in the temporal lobe and the fourth module was termed as the SP module (Fig. 2a). These modules were restricted to the left hemisphere and six other homotopic modules were generated for the right hemisphere as well using the AAL markers. The set of 12 modules so generated was then analyzed for FC changes.

The intermodular connections were found by summing the connectivity from each ROI of one module to that of the other module (Fig. 1b). The modular connections obtained for each subject in the group from each module with the other module are termed the modular connectivity matrix. For single group analysis, the modular connections are statistically tested against the alternative hypothesis of a nonzero mean using a *p* value of 0.05 using the *t*-test with unknown mean and unknown variance. For group comparison, the statistical test used was the *t*-test for two groups with unknown means and unknown but equal variances against the alternative hypothesis of different means with a significance value of 0.05. The statistical tests are not corrected for multiple comparisons.

The modular connectivity matrix was then analyzed for various conditions (such as ES, UR, and BL), intersubject group comparisons (such as test group > control group), and intercondition comparisons (such as UR > BL). The modular connectivity was plotted using BrainNet (Xia et al., 2013). The connectivity of the test, normal, and control groups was estimated during the different conditions such as UR, ES, and BL, and for the first and final sessions. Intergroup comparisons were performed for (A) normal group > test group for the first session, (B) test group > control group for the increase in FC in the final session over the first session.

The color bar on the right indicates the value of the FC between each pair of modules (red for positive values and blue for negative values of the sum of FC values of all pairs of ROIs, and the pair consisting of one ROI taken from the first module and the other from the second module). The FC value is obtained by first computing the correlation of the two ROIs' BOLD time series as given in Equation 2 and subsequently applying a Fisher transform. The following convention is used during the presentation of results: significance of the statistic (*t*) is presented with asterisks as *p* < 0.05 (*), *p* < 0.01 (**), *p* < 0.001 (***), and *p* < 0.0001 (****).

## Results

**FIG. 3.**
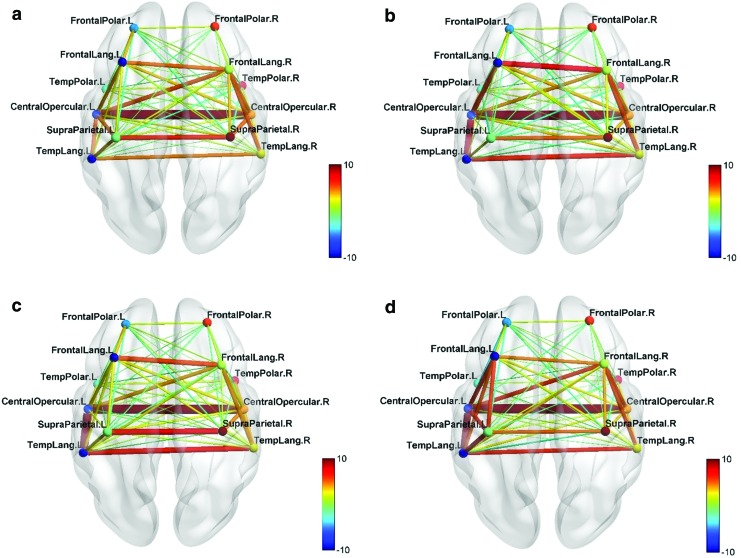
FC networks of test group over the four neurofeedback training sessions S2–S5 **(a–d)**. The connections in the left hemisphere between modules FL, TL, SP, and CO were observed to progressively strengthen with the number of sessions. The color bar indicates the strength of the intermodular connections. CO, central opercular; FL, frontal language; SP, supraparietal; TL, temporal language. Color images are available online.

**FIG. 4.**
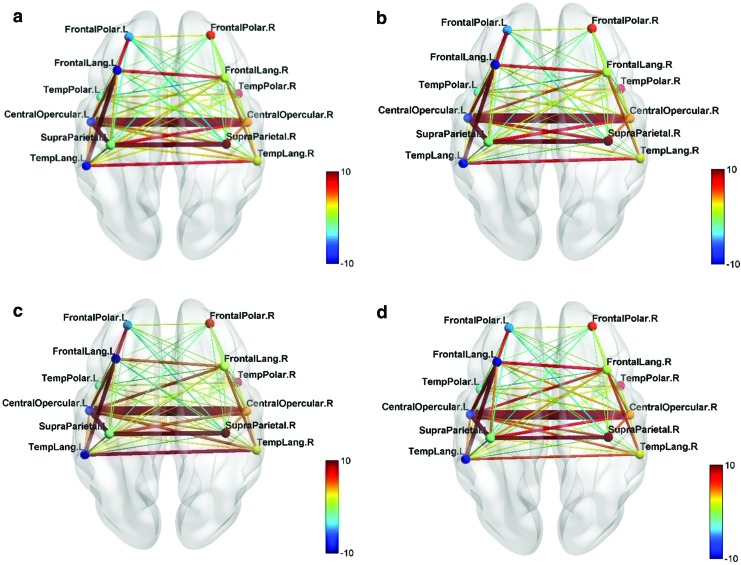
FC networks of normal group over the four neurofeedback training sessions S2–S5 **(a–d)**. The connections in the left hemisphere between modules FL, TL, SP, and CO are observed throughout the sessions. Only the connection between FL and CO modules was observed to strengthen during S5 **(d)**. Color images are available online.

The FC networks obtained among the modules during each of the training sessions are shown for test and normal groups in Figures 3 and 4.

With training, the functional connections in the left hemisphere for the test group were observed to strengthen among the modules FL, TL, SP, and CO. Notably, a positive connection was observed between FL and TL modules. For the normal group, connections were observed in the left hemisphere and the strengthening was not as pronounced as for the test group.

**FIG. 5.**
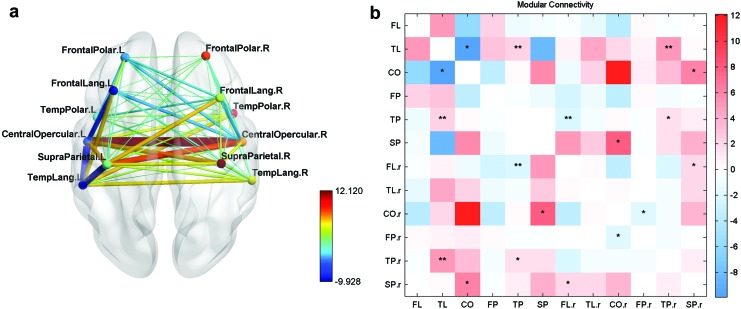
**(a)** Normal group > test group—S1. **(b)** Modular connectivity matrix. FC networks and intermodular connections of normal group compared with test group during first session (Comparison A) *p* < 0.05 (*), *p* < 0.01 (**); the bar in red and blue on the right indicates the strength of the intermodular connections between each pair of modules on the *x* and *y* axes. Color images are available online.

**FIG. 6.**
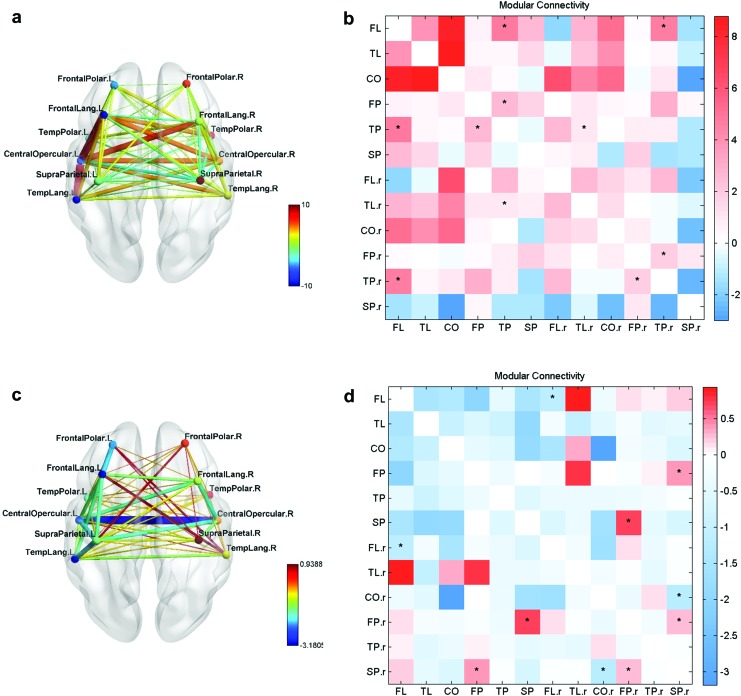
**(a)** FC for 2nd half >1st half—normal group. **(b)** Modular connectivity matrix. **(c)** FC for UR > BL—normal group. **(d)** Modular connectivity matrix. **(a, b)** FC networks and intermodular connections of normal group—2nd half of sessions compared with 1st half (Comparison F). **(c, d)** FC networks and intermodular connections of normal group during upregulation compared with the baseline condition (Comparison E). *p* < 0.05 (*); the bar in red and blue on the right indicates the strength of the intermodular connections between each pair of modules on the *x* and *y* axes. Color images are available online.

For the normal group during intergroup comparison (A) normal group > test group, it was observed that the FC was high between modular pairs CO–CO.r, FL–TL, and SP–CO (Fig. 5a). For the intragroup comparison (F) second-half > first-half for the normal group, it was seen that the FC was high between modular pairs FL–CO and CO–TL. This shows that these connections have strengthened during the neurofeedback training and they were mainly strengthening in the left hemisphere (Fig. 6a, b). However, these connections were not significantly positive.

For the intragroup comparison (E) UR > BL for the normal group, there were only minimal changes in the FC network (Fig. 6c, d). Modules FL and FP were connected to the right hemispheric modules of SP and TL and left hemispheric connections showed no increase during the upregulation task.

**FIG. 7.**
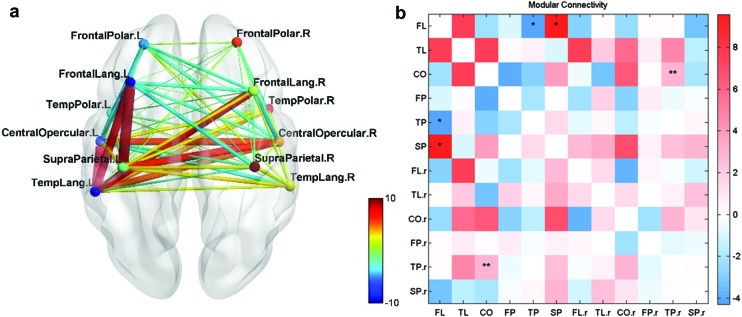
**(a)** FC for 2nd half >1st half—test group. **(b)** Modular connectivity matrix. FC networks and intermodular connections of test group—2nd half of sessions compared with 1st half (Comparison D). *p* < 0.05 (*), *p* < 0.01 (**); the bar in red and blue on the right indicates the strength of the intermodular connections between each pair of modules on the *x* and *y* axes. Color images are available online.

For the intragroup comparison, (D) second-half > first-half for test group, the increase in FC is highest for modular connections FL–SP, FL–TL, and TL–CO. This shows that the connections are strengthening during training mainly in the left hemisphere (Fig. 7).

In particular, the FL–SP connection is significantly higher during the latter half of the sessions. Furthermore, a direct connection between the FL and TL modules was observed though not significantly greater from that for the former half of sessions.

**FIG. 8.**
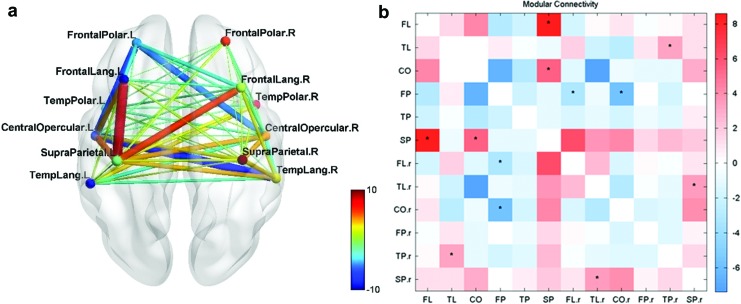
**(a)** Test group > control group and final session > first session. **(b)** Modular connectivity matrix. FC networks and intermodular connections of test group over control group during the final session over the first session (Comparison B). *p* < 0.05 (*); the bar in red and blue on the right indicates the strength of the intermodular connections between each pair of modules on the *x* and *y* axes. Color images are available online.

To study the effect of neurofeedback training an intergroup comparison, (B) test group > control of the final session over the first session was performed. This showed that FC is highest for modular pairs FL–SP on the left hemisphere (Fig. 8). Less strong connections are seen between modules FL–CO and CO–SP. Thus, it can be inferred that neurofeedback training has strengthened primarily left hemispheric connections as was seen in intragroup comparison (D) earlier and the FL–SP connection is most pronounced.

**FIG. 9.**
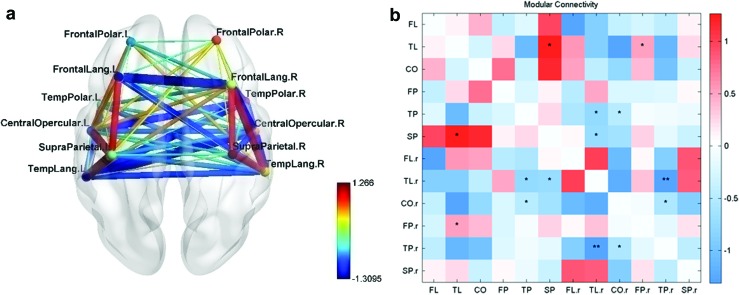
**(a)** Test group—UR > BL. **(b)** Modular connectivity matrix. FC networks and intermodular connections of test group during upregulation compared with the baseline condition (Comparison C). *p* < 0.05 (*), *p* < 0.01 (**); the bar in red and blue on the right indicates the strength of the intermodular connections between each pair of modules on the *x* and *y* axes. BL, baseline; UR, upregulation. Color images are available online.

For the intragroup comparison (C) UR > BL for test group, there are changes in the FC network in both hemispheres. The connections improve between modules FL–SP, CO–SP, and TL–SP on the left and modules FL.r–TL.r, FL.r–SP.r, and SP.r–TL.r on the right hemisphere (Fig. 9). Strong connections are observed during upregulation in the left hemisphere when compared with the baseline condition, with an indirect connection between FL and TL modules through the SP module, indicating that the upregulation exercises the left temporofrontal network and restores connections through alternative pathways.

The left SP module has a larger number of strong connections within the left hemisphere. An indirect connection from left FL to TL modules is seen through the SP module. Thus, with upregulation, it is seen that the SP module plays a central role in the recovering connectivity network on the left hemisphere. The results also show that the right hemisphere has connections during the upregulation task between modules FL.r and SP.r and between modules SP.r and TL.r and notably directly between FL.r and TL.r modules.

During the intragroup comparison, (G) final session over the first session during the baseline condition, it is seen that the FL and SP modules connect strongly. The SP module also connects to the FL.r and CO.r modules on the right hemisphere.

**FIG. 10.**
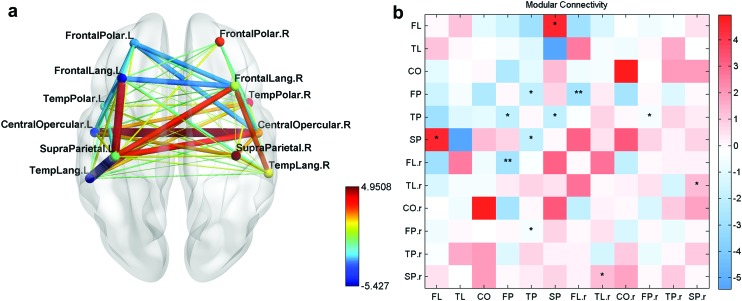
**(a)** Test group—S6 > S1 during BL. **(b)** Modular connectivity matrix. FC networks and intermodular connections of test group during final session compared with first session during baseline condition (Comparison G). *p* < 0.05 (*), *p* < 0.01 (**); the bar in red and blue on the right indicates the strength of the intermodular connections between each pair of modules on the *x* and *y* axes. Color images are available online.

The SP module connects to the FL and TL modules in several of the mentioned comparisons (B, C, D, and G) with a significance of *p* < 0.05, thus indicating a strengthening of an alternative pathway between the FL and TL modules through the SP module. The FL–SP connection was significantly strengthened even in the baseline condition when the test group was at rest as shown in Comparison G (Fig. 10), indicating that the neurofeedback training was able to strengthen even when the patients were not upregulating or engaging in language activity.

## Discussion

FC changes due to the RT-fMRI-based neurofeedback training have been analyzed in stroke patients with expressive aphasia. The hypothesis was that the neurofeedback training would enhance the FC between the language regions of the brain and restore it toward normalcy. RT-fMRI in addition to providing neurofeedback also gives a time series of brain images that can be analyzed for FC changes. Cortical and subcortical structures can be accurately delineated and studied through this approach.

The stroke infarct has weakened the left hemispheric connections of the test group between the FL and TL modules as well as between the left and right CO modules when compared with the normal group. With increasing sessions, it can be seen that these connections were strengthening and tending toward normalcy. The neurofeedback training has strengthened the left hemispheric connections between the FL and SP modules. The left hemispheric connections were stronger for the test group than for the control group and could be attributed to the training. With upregulation for the test group, FL and TL modules connect indirectly through the SP module. It can be inferred that the SP module plays an important role in the recovering language network. The SP module connects strongly with the FL module even during the baseline condition, showing a persistent effect of the neurofeedback training.

When changes with sessions were measured within the test group, left hemispheric connections have strengthened more between FL and SP modules. In addition, when comparing the latter half with the former half of sessions, a direct connection was observed from FL to TL modules. This shows that the training induces recovery mainly by strengthening connections in the left hemisphere. Further weaker connections are observed between FL–TL and TL–CO modules. This shows that with training over sessions, there were several connections strengthening in the left hemisphere involving the Wernicke's area, angular gyrus, and other regions in the left perisylvian area. One of the objectives in providing a neurofeedback signal that was amplified by the correlation between the two ROIs in the IFG and STG was to improve the connectivity between these regions. The strengthening of the FL–TL connections has shown that this approach was successful in doing so.

During intragroup contrast of upregulation over baseline, connections in both the hemispheres were observed to strengthen. Right hemispheric connections were also observed to strengthen between the right FL and TL modules, as well as between the right TL and right SP module. However, the increase is only one-third of FC changes when compared with other intergroup comparisons. This shows that during upregulation, there was a slight strengthening of the left and right language network involving the FL, TL, and SP modules.

The grouping of several regions into modules and analyzing the connectivity trends between modules are visually easier than analyzing individual ROI-to-ROI connections. The statistical significance of the sum of Fisher connectivity scores between modules has also been analyzed by calculating each subject's connectivity scores during each condition or comparison and finding out the intragroup or intergroup variance of the means. To assess the statistical significance of the modular connectivity, a *t*-test is performed with a significance level set to 0.05. This technique gives a panoramic view of the connectivity at a coarser scale of resolution. There are concerns that the positive and negative connections may be significant in themselves; however, the sum may be low and indicate a near-zero connection strength, and too many small negative connections can cancel out strong connections during the summation and result in near-zero connections or *vice versa*. However, these concerns could be minor if the ROIs of a module are close neighbors as in this study and the modularity statistic ensures similarity among the ROIs.

fMRI and FC studies have revealed that other areas in the left temporofrontal cortex also play an important role in language (Price, 2010; Tomasi and Volkow, 2012). A detailed review of several positron emission tomography (PET) and fMRI studies found that multiple regions surrounding Broca's and Wernicke's areas are involved in semantic association and retrieval, articulatory association and sequencing, processing of auditory or visual language stimuli, and generation of speech (Price, 2012). Our study has shown that several of these regions grouped as modules were connected strongly during the training. The SP module, consisting of the precentral and postcentral gyri, anterior regions of the supramarginal gyrus, and the SP lobule, increasingly connects to the FL module and CO module in the left hemisphere with training. Furthermore, the SP module also connects to the CO module and Wernicke's area during upregulation, though less strongly. A direct pathway during the second half of the sessions is seen connecting the left FL module and the left TL module as well as the left FL module and the SP module. The expression of speech being processed in the residual Broca's area was being aided by the formulation of speech in the Wernicke area (semantic association area in the temporal lobe) so that the upregulation tends to promote production of meaningful language.

It is instructive to consider the aims and results of our study in the context of extant empirical research and theoretical understanding of language processing in health and disease. An earlier model of language processing in healthy humans that was widely accepted by neurologists was the single-route model by Geschwind (1965a,b, 1979). According to this model, a heard word is first converted into phonemes in the primary auditory cortex after which semantic associations are formed in the Wernicke's area, which finally leads to a motor output through activations in the Broca's area and the supplementary motor cortex. The model proposes that, although a visual word is phonologically processed in the angular gyrus first, it nevertheless forms semantic associations in the Wernicke's area and the motor output in the Broca's area, the latter processes being similar to those involved in heard words. Hence, this model proposes a predominantly unitary path for both heard and read words.

The mentioned model was contested by the multiple-route model by a number of proponents (Coltheart et al., 1987; LaBerge and Samuels, 1974; Rumelhart et al., 1986), who proposed distinct and parallel paths for phonological and semantic processing. A functional imaging study using PET by Petersen et al. (1988) showed inconsistencies with the single-route model, which led the authors to propose the first functionally and anatomically relevant multiple-route model. In this model, auditorily and visually presented words follow parallel and somewhat independent trajectories in lexical and semantic processing until motor output is generated. A distinction of this model from the single-route model is that visual words do not establish semantic associations in the Wernicke's area but in the prefrontal cortex before motor output. A further distinction is that sensory information extracted from seen and heard words may independently lead to separate semantic and motor processes, as observed in the lack of activation of semantic association areas to the repetition of words by the participants. Following the mentioned model and based on numerous PET and fMRI studies on language processing in the past two decades, a greater understanding of language processing has resulted, culminating in the anatomically more precise and functionally refined multiple-route model (see figure 2 of Price, 2012).

In light of the mentioned healthy model of lexical and semantic processing, a question then arises as to how we could explain the effect of stroke and its recovery in language processing in aphasia patients. Functional imaging studies in aphasia patients have shown evidence for increased activity in the perilesional and homologous contralesional areas. An fMRI study of aphasia patients with repeated measurements identified neutrally and behaviorally three distinct phases of recovery (see figure 5 of Saur et al., 2006). In the acute phase (mean 1–2 days poststroke, mdps), speech was noticeably disrupted, and activation of the IFG was considerably weakened in comparison with the homologous area in the right hemisphere. In the subacute stage (12 mdps), a large activation in the right IFG but not a comparable increase in the left IFG was observed, and the increase in regional activations was strongly correlated with recovery in language function. In the chronic phase of recovery (320 mdps), a return to normal activation in the left IFG, a reduction in activation in the right IFG, and a further recovery in language function were observed. Considering the mentioned pattern of recovery after stroke in aphasia, future neuromodulation studies may benefit from mimicking the mentioned pattern of brain activation in the affected and homologous regions of the language network pertaining to the chronicity of the stroke in the patient.

Our study, which was performed in the subacute phase, shows that the left hemispheric FC increased after training. The FC among peri-infarct left language areas was increasing and may possibly consolidate to the normal with additional training and language recovery. Further evidence in this regard was seen during upregulation when the left FL module and the left TL module are indirectly connected, though weakly. The part of this indirect connection between the FL and SP modules was significantly strengthened even during the baseline condition when the patients were at rest.

Longer and more intense neurofeedback training may reveal the progression of the observed connectivity changes as well as their role in restoring language function. The feedback signal from Broca's^†^ area was modified by a correlation between it and Wernicke's^†^ area. A recent review by Watanabe et al. (2017) discusses the use of connectivity-based feedback and multivariate feedback for precise manipulation of spatiotemporal brain activity patterns and its clinical applications. This feedback should in principle also increase the connectivity between these areas and was evidenced by the increased connectivity between FL and TL modules during comparison of the latter half with the former half of the training sessions.

## Limitations of the Study

The sample size of our study was small. Our initial target was a set of six test and six control patients. This had to be reduced to four each due to the low number of patients who could be screened in and further cooperate for the full duration of the study. Although the patients are diagnosed Broca's aphasic, there are differences in the underlying lesion size and extent. There are differences among the patients regarding the severity of the stroke as well as the interval between recruitment and ictus. Elderly patients were less likely to undergo all the training sessions, whereas the younger patients were more likely to undergo all the six sessions of the RT-fMRI neurofeedback. The interval between the pretest and post-test sessions should also be ideally the same for all the test and control patients. The recruitment of patients and their progress during the RT-fMRI training could also be mapped as a CONSORT flow diagram. It could be argued that the connectivity changes observed were not due to neurofeedback *per se*, but rather due to the repeated language tasks. However, by carefully redesigning the experiment, neurofeedback-specific responses and neurofeedback nonspecific responses (dependent on the neurofeedback context, but independent from the act of controlling a particular brain signal) can be separated. Since no language improvement was observed, further research is required to identify the role of the substrates activated and the connections strengthened in inducing language recovery. More intense neurofeedback training may be necessary to induce measurable recovery outcomes, which could be implemented using training strategies with less cumbersome and less expensive neurofeedback mechanisms such as electroencephalogram or functional near infrared spectroscopy. Development of a language task sensitive to even subtle changes in language performance due to the neurofeedback training could be another aspect of further research.

Future work could consider patients with matched lesions, an increased duration and intensity of the neurofeedback training, and a larger patient group. The Louvain algorithm for partitioning the connectivity matrix into modules did not always give the same or similar partitions. The algorithm could be modified so as to improve the repeatability of the partitions. The intensity of the connections within a single module could also be studied for changes with neurofeedback training. This would indicate the modules that strengthen in terms of internal connections and respond to the neurofeedback training. Data-driven approaches for FC such as independent component analysis can be employed as well as the ROI set expanded to include the whole cerebral cortex to enable a complete view of the residual language substrates recruited with training and the dynamic interplay of the connections between them.

## Conclusion

The FC analysis of the RT-fMRI data shows that post-training the connections are stronger in the left hemisphere for the test group that for the control group. The stroke infarct has weakened the left hemispheric connections of language areas for the test group when compared with those of the normal group. The SP module is strongly connected to the FL module with more training sessions and the TL module during upregulation, and plays an important role in the recovering language network. Neurofeedback training shows the strengthening of left hemispheric connections, including a direct connection between the left FL and left TL modules, and is restoring the connections toward that of the normal group. The change in the connectivity among modular regions of the brain has been delineated during RT-fMRI neurofeedback. The residual language networks being exercised poststroke were visualized and future intervention using neuromodulation can be targeted to strengthen these residual networks and constituting regions for restoring language function.

## Supplementary Material

Supplemental data

## Footnotes

^†^ Including perilesional areas as identified from the functional localizer.
