# Supplementary material for: Functional Connectivity of Language Regions of Stroke Patients with Expressive Aphasia During Real-Time Functional Magnetic Resonance Imaging Based Neurofeedback
Source: Brain Connect. 2019 Oct 17;9(8):613–26. doi: 10.1089/brain.2019.0674 (PMC6798872; doi:10.1089/brain.2019.0674)
Supplement: Supplemental data [file Supp_Data.pdf]

Supplementary Data

Inclusion Criteria

The criteria for patient recruitment are summarized in Supplementary Table S1.

Functional Localizer

The functional localizer was performed in every session to identify the target regions of interest (ROIs) to be used for neurofeedback training. The functional localizer involved a sequence of five-word generation tasks, each task consisting of a letter from the Malayalam alphabet presented visually. The subject had to continuously generate different words starting with that letter for 10 scans for a duration of 15 s. Each word generation task was separated by a rest block having a duration of 15 s. The functional localizer was processed by the Turbo Brain Voyager in real time, and the significantly activated clusters were generated immediately after the functional localizer run. The cluster in and around the Broca's area (inferior frontal gyrus [IFG] pars triangularis, IFG pars opercularis) was selected as ROI_1 and the cluster in and around the Wernicke's area (pSTG) was selected as ROI_2.

Supplementary Figure S1 shows the architecture of the real-time functional magnetic resonance imaging neurofeedback system. During the baseline condition, a constant level of 10 blue bars is shown visually on a blue background. The blue background is a cue to the subject to rest during the baseline condition. During the upregulation condition, the background color of the visual feedback changes to green, providing a cue to the subject to engage in language tasks and increase the level of the thermometer. Increases above the baseline are shown as additional red bars and for decreases below the baseline, the blue bars are removed proportionately.

The selected ROI_1 and ROI_2 are shown in Supplementary Tables S2 and S3, respectively, for the test patients during the six sessions of neurofeedback pretest, training, and post-test sessions. The coordinates of the center of each ROI and its volume are reported hereunder.

The ROI coordinates as well as volumes are reported in the Montreal Neurological Institute space.

SUPPLEMENTARY TABLE S1. INCLUSION AND EXCLUSION CRITERIA

Inclusion criteria	*Exclusion criteria*
Patient with age >18 years, diagnosed with expressive aphasia, and within the interval of 6 weeks to 6 months poststroke	Patients having receptive aphasia
Right-handed according to Edinburgh handedness inventory	Having a pacemaker device or other metallic implants that are not MRI compatible
Willing to cooperate for the study	Claustrophobic
Motivated for speech therapy	Patients needing spectacles for short sightedness
Primary language should be Malayalam	

MRI, magnetic resonance imaging.

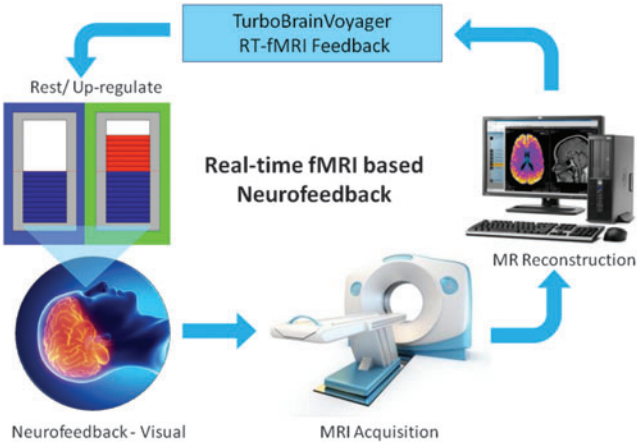

SUPPLEMENTARY FIG. S1. Architecture of a RT-fMRI-based neurofeedback system. RT-fMRI, real-time functional magnetic resonance imaging.

Supplementary Table S2. ROI_1 Corresponding to the Active Cluster in and Around the Broca's Area

	S1	*S2*	*S3*	*S4*	*S5*	*S6*	*Mean*	*SD*	*Location of mean*
T1 Broca									
X	−55	−51	−64	−62.6	−51.7	−49.6	−55.7	6.2	IFG
Y	20.2	21.8	12.6	26.7	11.7	5.5	16.4	7.8	Fronta_inf_Tri_L
Z	5.5	−4.8	10.8	8.1	4.5	3.3	4.6	5.3	
Vol	280	256	216	456	968	1080	542.7	383.4	
T2 Broca									
X	−41.2	−49.7	−48	−42.1	−50.6	−45.1	−46.1	3.9	IFG
Y	10.9	19.9	10.1	23.5	0.5	7.8	12.1	8.4	Fronta_inf_Oper_L
Z	23.7	34.4	18.5	0.3	−3.4	−5.7	11.3	16.5	
Vol	2112	248	2528	648	1560	1696	1465.3	867.1	
T3 Broca									
X	−46.4	−59.4	−56.3	−50.6	−53.5	−54.6	−53.5	4.5	IFG
Y	−7.7	−8.1	−3.5	18.4	17.5	1.4	3.0	12.1	Rolandic_Oper_L
Z	−2.9	15.2	12.8	2.2	−1.1	5.3	5.3	7.4	
Vol	1192	4200	2312	2008	1184	1488	2064.0	1139.6	
T4 Broca									
X	−59.1	−48.1	−48	−46.9	−49.2	−47.9	−49.9	4.6	IFG
Y	13.8	20.2	13.8	14.7	14.4	14.9	15.3	2.4	Fronta_inf_Oper_L
Z	5.2	0.6	−1.2	−4.2	−3.7	11.1	1.3	5.9	
Vol	2264	3112	2848	1416	2168	2000	2301.3	608.2	

SD, standard deviation.

Supplementary Table S3. ROI_2 Corresponding to the Active Cluster in and Around the Wernicke's Area

	S1	*S2*	*S3*	*S4*	*S5*	*S6*	*Mean*	*SD*	*Location of mean*
T1 Wernicke									
X	−41.9	−41	−55.7	−44.6	−59.9	−60.1	−50.53	9.0	STG
Y	−50.1	−50.5	−37.9	−57.3	−36.7	−43.2	−45.95	8.1	Temporal_Sup_L
Z	23.6	7.8	23.8	25	12.2	10.3	17.12	7.8	
Vol	856	1072	712	1312	608	600	860	283.6	
T2 Wernicke									
X	−43.6	−45.7	−41.7	−59.4	−53.9	−54.5	−49.80	7.1	STG
Y	−48	−36.1	−43.4	−45.7	−47.8	−43.4	−44.07	4.4	Temporal_Sup_L
Z	31.3	42.8	30	5.9	6.5	3.9	20.07	16.7	
Vol	368	168	672	648	888	2176	820	710.5	
T3 Wernicke									
X	−50.2	−57.1	−53.3	−61.4	−59.7	−49.6	−55.22	4.9	STG
Y	−55.9	−42.7	−41.3	−34.1	−38.8	−53	−44.3	8.4	Temporal_Sup_L
Z	2.6	15.3	12	7.6	12.8	8	9.72	4.6	
Vol	2104	2392	3800	656	2928	1992	2312	1047.7	
T4 Wernicke									
X	−61.5	−56.2	−60.2	−53.2	−62.2	−60.8	−59.0	3.5	STG
Y	−28.2	−48.5	−35.2	−49.9	−31.8	−48.2	−40.3	9.7	Temporal_Sup_L
Z	12.1	8.4	4.2	6.6	−1.3	4.4	5.73	4.5	
Vol	1928	1024	784	2344	3168	1040	1714.7	933.0	
